# One Digit Interruption: The Altered Force Patterns during Functionally Cylindrical Grasping Tasks in Patients with Trigger Digits

**DOI:** 10.1371/journal.pone.0083632

**Published:** 2013-12-31

**Authors:** Po-Tsun Chen, Chien-Ju Lin, I-Ming Jou, Hsiao-Feng Chieh, Fong-Chin Su, Li-Chieh Kuo

**Affiliations:** 1 Department of Biomedical Engineering, National Cheng Kung University, Tainan, Taiwan; 2 Medical Device Innovation Center, National Cheng Kung University, Tainan, Taiwan; 3 Department of Orthopedic Surgery, National Cheng Kung University, Tainan, Taiwan; 4 Department of Occupational Therapy, National Cheng Kung University, Tainan, Taiwan; The University of Queensland, Australia

## Abstract

Most trigger digit (TD) patients complain that they have problems using their hand in daily or occupational tasks due to single or multiple digits being affected. Unfortunately, clinicians do not know much about how this disease affects the subtle force coordination among digits during manipulation. Thus, this study examined the differences in force patterns during cylindrical grasp between TD and healthy subjects. Forty-two TD patients with single digit involvement were included and sorted into four groups based on the involved digits, including thumb, index, middle and ring fingers. Twelve healthy subjects volunteered as healthy controls. Two testing tasks, holding and drinking, were performed by natural grasping with minimal forces. The relations between the force of the thumb and each finger were examined by Pearson correlation coefficients. The force amount and contribution of each digit were compared between healthy controls and each TD group by the independent *t* test. The results showed all TD groups demonstrated altered correlation patterns of the thumb relative to each finger. Larger forces and higher contributions of the index finger were found during holding by patients with index finger involved, and also during drinking by patients with affected thumb and with affected middle finger. Although no triggering symptom occurred during grasping, the patients showed altered force patterns which may be related to the role of the affected digit in natural grasping function. In conclusion, even if only one digit was affected, the subtle force coordination of all the digits was altered during simple tasks among the TD patients. This study provides the information for the future studies to further comprehend the possible injuries secondary to the altered finger coordination and also to adopt suitable treatment strategies.

## Introduction

Trigger digit (TD) is a kind of stenosing tenosynovitis, and a common hand disorder in orthopedic or hand clinics. The maximal grip strength is generally used as an indicator to evaluate the severity [1] or treatment effect [2], [3] of patients with TD. In addition to the power grip of hand, subtle coordination among the applied forces of the digits is also critical in various daily activities. For example, when drinking from a bottle of water, the digits must apply the proper forces to grasp the bottle without squeezing it or letting it slip, and then raise it so that the water pours smoothly into the mouth. However, not only the specific force patterns during such daily grasping functions remain unknown, but also the impact of finger disturbances, such as TD.

Researchers have studied the force patterns in various experimental conditions to investigate how forces of fingers are coordinated, especially with regard to different locations of the center of mass or external applied torque, by utilizing apparatus with five force transducers [4]–[6]. In this way the role of each digit can be discovered, such as the index finger serves as the agonist to provide pronation torque in a pouring task. Another study investigated the effects of finger addition or removal on multi-finger prehensile performance during a jar holding activity [7]. In addition, the digit force patterns were also found to be altered in patients with impaired tactile sensation [8]. Although the impacts of TD have been shown in a number of clinical evaluations [1]–[3], it remains unknown whether this condition affects the subtle force coordination among digits during hand manipulations. Clinical experience also indicates that TD is seen as a condition in which function-related issues regarding hand performance tend to be neglected, due to the lack of any apparent trauma or change in appearance of the hand.

In fact, most TD patients complain that they have problems using their hand due to single or multiple digits being affected. Unfortunately, clinicians do not know much about such problems, because few studies have examined the impact of the single- or multi-digit TD-related disturbances on the hand coordination and functional hand performance and then provide sufficient evidences to make suitable intervention plans. To understand the influence of TD on force kinetics or coordination of the digits, more specific equipment or experimental setups are needed, rather than simply the use of conventional grasp power measurements. To achieve this, a cylindrical simulator has been developed [9] to detect the digit forces that occur during cylindrical grasping which is the most common hand function that requires the use of all five digits in daily life. Consequently, the purpose of present study was to investigate applied forces by all five digits in patients with TD and healthy controls (HC). This study hypothesized that the patients with TD would exert less force on the involved digit than the HC do during the cylindrical grasp; in addition, the reduced force correlation between the involved digit and the others during the five-digits grasping task in the TD patients might be demonstrated in this experimental setup. Likewise, the intact digits may exert forces in a compensatory pattern with regard to the involved digit. More specifically, the force coordination patterns were analyzed during functional holding and drinking task by natural grasping with all five digits, and the changes in the kinetic performances due to different digits affected during the cylindrical grasping are reported in this study.

## Materials and Methods

### Participants

Forty-two patients who were diagnosed with idiopathic TD participated in this study. All of them were referred by the same physician after a physical evaluation and sonographical examination in the Department of Orthopedics at National Cheng Kung University Hospital (NCKUH). Patients with single digit involvement only were included in the study, and assigned to one of four groups according to the involved digit (Table 1). Twelve healthy controls with ages ranging from fifty to seventy years old volunteered as the healthy control group (HC, one male and 11 females, 60.0±12.0 years). The participants were excluded if they were diagnosed with multi-digit TD, carpal tunnel syndrome, diabetes, any neurological sign or other sensory disorders, or musculoskeletal disorders of the wrist and hand at the measured side that would affect grasping performance.

**Table 1:** Basic data of each TD group sorted by involved digit.

				Impairment grade of trigger digit		
Group	Affected digit	Age (years)	Number	Grade II	Grade III	Grade IV
TD_T_	thumb	55.7±7.6	10 (female: 10)	0	3	7
TD_I_	index finger	61.5±3.5	7 (female: 3)	1	1	5
TD_M_	middle finger	57.7±8.8	15 (female: 10)	2	4	9
TD_R_	ring finger	57.9±9.4	10 (female: 6)	0	3	7

### Ethics Statement

All participants were informed about the purpose of the study and signed consent forms. The study was approved by the Institutional Review Board (No. HR-98-048) of National Cheng Kung University Hospital.

### Instruments

**Figure 1:**
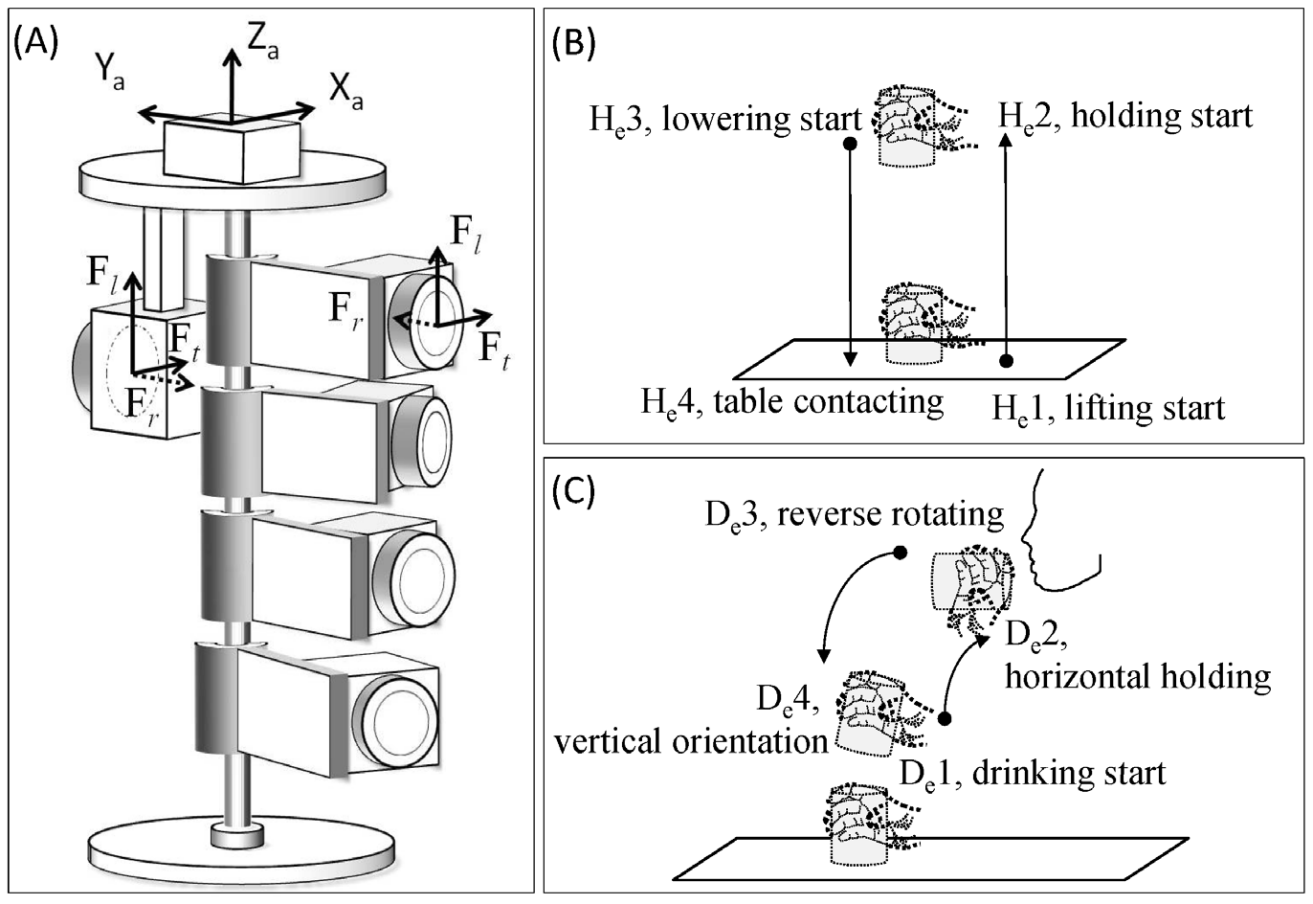
The cylindrical simulator and functional tasks. (A) Diagram of the cylindrical simulator, showing five force/torque transducers, the accelerometer and their local coordinate systems. Based on the obtained acceleration data, thus four movement events in the (B) holding and (C) drinking tasks were determined.

A custom cylindrical simulator (Fig. 1A) was designed with five force/torque transducers mounted on it (Nano-25 and Nano-17s, ATI Industrial Automation, Apex, NC, USA) to record the applied digit forces. One transducer (Nano-25) was for the thumb, and the other four transducers (Nano-17) were for the four fingers. The force transducers could be adjusted according to each participant’s grasping configuration and hand size. A tri-axial accelerometer (Silicon Designs, Inc., USA) was also attached to measure the acceleration of the simulator while the tasks were carried out. The force transducers and accelerometer were connected to an analog/digital data acquisition system (GW Instruments, Inc., USA) in order to digitize the data synchronously at a rate of 1000 Hz.

The local force coordinate system of each transducer (Fig. 1A) was selected as the radial force in the radial direction (F*_r_*) with respect to the simulator, the tangential force in the longitudinal direction (F*_l_*), and the tangential force in the transverse direction (F*_t_*). The resultant force (*RF*) was the resultant of orthogonal components of applied force. The *RF* and the components of F*_r_* and F*_l_* were used in the following analysis.

### Experimental Procedure

Before the experiment began, the participants were asked to clean their digit pads with alcohol swabs. The resting position of each participant was sitting upright on a height adjustable chair with the upper arms against the side of body and forearms resting on the table in front of them. The simulator was aligned with the midline of the participant at a distance equal to the length of their forearm. Participants were asked to grasp the simulator in their usual way. The experimenter adjusted the positions of the transducers so that each digit pad was in contact the center of corresponding transducer. The participants were told how to carry out the two testing tasks, and to use minimal force, and asked to familiarize themselves with both the apparatus and tasks. After hearing the verbal cue of “start”, each participant began to perform the trial at a self-selected performing speed, and was able to restart if any error occurred. Three successful trials of each task were required.

For the holding task (Fig. 1B), the participants grasped the simulator and lifted it upward to the height of 20 cm, held it in this position for at least for 3 seconds, and finally lowered it to the original place. During the drinking task (Fig. 1C), the participants grasped and moved the simulator towards the mouth and rotated it about 90° in the direction of pronation, and then reversed the movement sequence returned the simulator backed to its original position on the table.

### Data Analysis and Statistics

**Figure 2:**
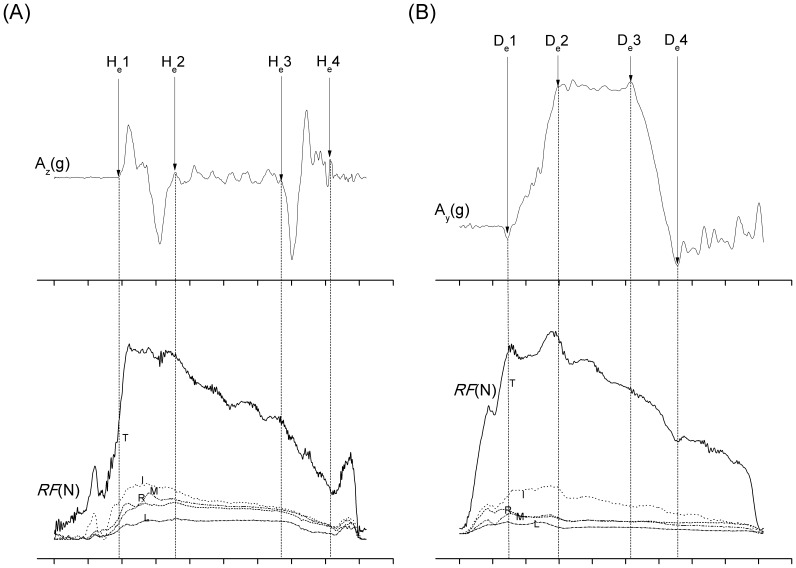
The determination of four movement events in each task. The events were found by the specific turning points of acceleration (A) along Z_a_ axis during the holding task including H_e_1 (the first turning point of the first positive peak of acceleration), H_e_2 (the first point of the sustained plateau which follows a negative peak), H_e_3 (the turning point at the end of plateau which is followed by a negative peak) and H_e_4 (the final peak), and (B) along Y_a_ axis by determining the four turning points (D_e_1, D_e_2, D_e_3 and D_e_4) of on trapezoid-shaped profile. Three phases were then defined in between sequential events in each task.

According to the turning points of acceleration, the movement sequences of each task could be separated into three phases based on the determination of four specific movement events (Fig. 2). The correlations between the F*_r_* as well as the F*_l_* of the thumb and each finger were calculated to show the relation of each thumb-finger pair [9]. The comparisons of each digital *RF* between the HC and TD groups were made to assess the differences in applied force. The contribution ratio (*CR*) was computed as each digital *RF* divided by the summed *RF* of five digits, and this represented the force distribution pattern among five digits.

The force correlation and *RF*, as well as *CR* of each digit, were analyzed at each event and during the second phases of both tasks. The force at the final event of holding task (H_e_4) was not discussed due to the simulator contacting with table. The Pearson correlation coefficient was used to examine the correlations of forces between the thumb and fingers of each group. However, the F*_l_* varied apparently within and between groups in both tasks, and there were seldom significant correlations or consistent patterns between the thumb and each finger. Since the emphasis was placed on the effect of a single triggering digit on the force pattern of all the digits, the independent *t* test was conducted to measure the differences in force variables between the TD and HC groups. All the statistics were calculated with the SPSS 17.0 (SPSS Inc., Chicago, Illinois, USA) software. The statistical significance was set at a level of *p*<0.05.

## Results

No participant complained about discomfort or the occurrence of triggering in the involved digits when performing the tasks.

### Force Correlation

The patterns of F*_r_* correlation between the thumb and each finger in the TD groups were different from that seen in the HC group (Table 2). In the results of the holding task for the HC group, the F*_r_* of the thumb showed significant correlations with that of index, middle and ring fingers. In the TD_I_ group, the thumb correlated significantly with index and little fingers. In TD_R_, the thumb correlated significantly with the index and middle fingers, as well as with the little finger. The results for both TD_T_ and TD_M_ groups showed that the thumb correlated significantly with all fingers across all the events. When the HC group performed the drinking task, the F*_r_* of the thumb correlated significantly with that of index, middle and ring fingers most of the time. The results for the TD_T_ group showed that the F*_r_* of the thumb and each finger was correlated significantly throughout the task. As for TD_I_, significant correlation of F*_r_* was found between the thumb and index finger. In the TD_M_ group, significant correlations were seen between the thumb and the index, middle and ring fingers throughout the task. While in TD_R_ group, the thumb and index finger showed a significant correlation.

**Table 2:** Correlation coefficients between the radial (Fr) of the thumb and each of the fingers during holding and drinking tasks by HC and each TD group.

		*Holding*					*Drinking*			
Group		T-I	T-M	T-R	T-L		T-I	T-M	T-R	T-L
**HC**	**H_e_1**	**.88**	**.84**	**.64**	.32	**D_e_1**	**.93**	.54	**.79**	.52
	**H_e_2**	**.87**	**.78**	.52	.36	**D_e_2**	**.87**	**.74**	**.80**	.27
	**H_ph_2**	**.87**	**.80**	.57	.43	**D_ph_2**	**.90**	**.80**	**.76**	.22
	**H_e_3**	**.88**	**.84**	**.63**	.53	**D_e_3**	**.83**	**.78**	**.71**	.19
						**D_e_4**	**.78**	**.71**	**.59**	.33
**TD_T_**	**H_e_1**	**.93**	**.95**	**.96**	**.91**	**D_e_1**	**.92**	**.89**	**.84**	**.71**
	**H_e_2**	**.94**	**.84**	**.97**	**.91**	**D_e_2**	**.88**	**.98**	**.84**	**.64**
	**H_ph_2**	**.94**	**.88**	**.97**	**.90**	**D_ph_2**	**.91**	**.96**	**.84**	**.66**
	**H_e_3**	**.92**	**.90**	**.97**	**.89**	**D_e_3**	**.93**	**.97**	**.87**	**.74**
						**D_e_4**	**.93**	**.98**	**.81**	.58
**TD_I_**	**H_e_1**	**.94**	**.76**	.58	.60	**D_e_1**	**.84**	.34	.18	**.76**
	**H_e_2**	**.91**	.57	.17	**.95**	**D_e_2**	**.81**	.47	.45	.61
	**H_ph_2**	**.92**	.58	.20	**.99**	**D_ph_2**	**.82**	.53	.37	.52
	**H_e_3**	**.90**	.59	.45	**.99**	**D_e_3**	**.84**	.47	.28	.64
						**D_e_4**	**.81**	.40	−.23	.51
**TD_M_**	**H_e_1**	**.84**	**.71**	**.89**	**.75**	**D_e_1**	**.64**	**.64**	**.92**	**.55**
	**H_e_2**	**.68**	**.64**	**.89**	**.76**	**D_e_2**	**.79**	**.67**	**.89**	.46
	**H_ph_2**	**.67**	**.65**	**.86**	**.70**	**D_ph_2**	**.80**	**.59**	**.83**	.42
	**H_e_3**	**.65**	**.64**	**.83**	**.64**	**D_e_3**	**.80**	.50	**.81**	.41
						**D_e_4**	**.84**	**.58**	**.91**	**.60**
**TD_R_**	**H_e_1**	**.88**	**.73**	.33	**.80**	**D_e_1**	**.77**	.16	.34	.30
	**H_e_2**	**.89**	**.66**	.35	.63	**D_e_2**	**.76**	.13	.56	.19
	**H_ph_2**	**.86**	.62	.48	**.67**	**D_ph_2**	**.71**	−.10	**.71**	−.04
	**H_e_3**	**.83**	**.63**	.57	**.69**	**D_e_3**	.62	−.25	.54	−.06
						**D_e_4**	**.82**	.22	**.67**	.27

Numbers in **bold** indicate statistical significance (*p*<0.05). T, I, M, R and L mean thumb, index finger, middle finger, ring finger and little finger.

### Applied Resultant Force

**Figure 3:**
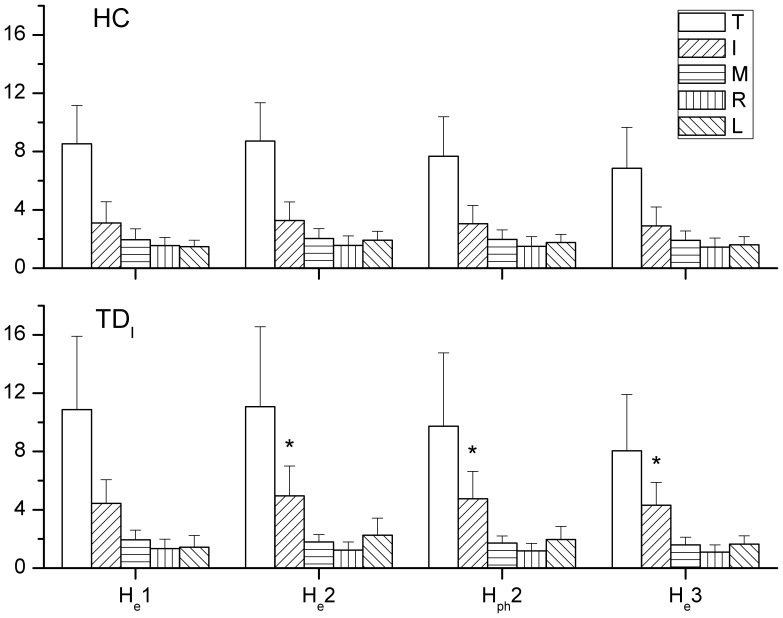
The significant difference of applied RF between TD_I_ and HC during holding. The *RF* of each digit at all events and the averaged *RF* during second phase of the holding task by HC (*upper*) and TD_I_ (*lower*). Larger forces of the index finger were found at H_e_2 and H_e_3 and during second phase for TD_I_ when compared to those for HC. Significant differences are indicated by * (*p*<0.05).

**Figure 4:**
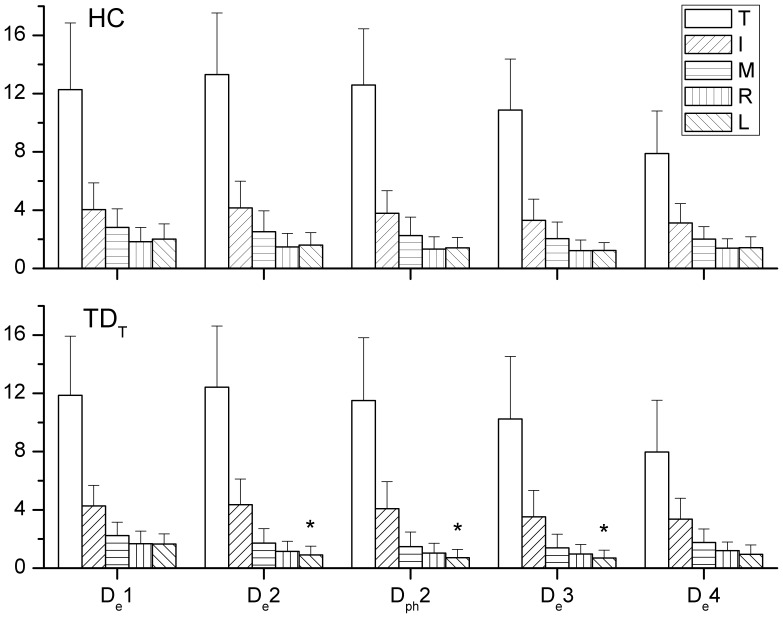
The significant difference of applied RF between TD_I_ and HC during drinking. The *RF* of each digit at all events and the averaged *RF* during second phase of the drinking task by HC (*upper*) and TD_T_ (*lower*). Lower forces for the little finger were found at D_e_2 and D_e_3 and during second phase for TD_T_ than those for HC. Significant differences are indicated by * (*p*<0.05).

The results of the independent *t* test revealed that larger *RF* by the index finger of patient in TD_I_ group at H_e_2 and H_e_3, as well as during the second phase of holding task, than that in HC (Fig. 3). During the drinking task, a lower *RF* of the little finger in TD_T_ than that in HC was seen at D_e_2, through the second phase, and at D_e_3 (Fig. 4). In addition to compare the *RF* of each digit, the total grasping force by summing the *RF* of all digits showed no significant difference between HC and each TD group during both tasks.

### Force Distribution Pattern

**Figure 5:**
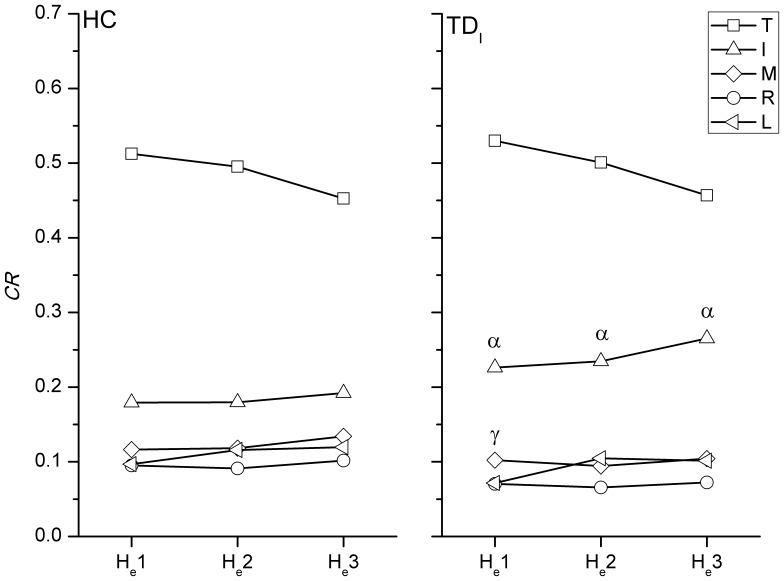
The significant difference of *CR* between TD_I_ and HC during holding. The force contribution ratios of each digit during the holding task in HC and TD_I_. A higher CR of the index finger and lower CR of the ring and middle fingers were noticed in TD_I_. Significant differences (p<0.05) in CR with regard to specific digits between HC and TD groups are indicated by the following symbols: α means the index finger and γ means the ring finger.

**Figure 6:**
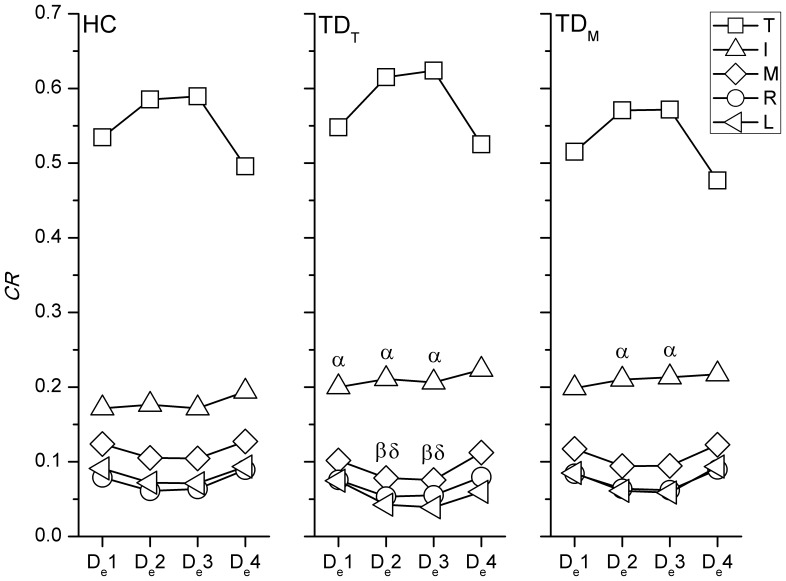
The significant difference of *CR* between TD_I_ and HC during drinking. The force contribution ratios of each digit during the holding task in HC, TD_T_ and TD_M_. A higher *CR* of the index finger and lower *CR* of the middle and little fingers were noticed in TD_I_ and TD_M_. Significant differences (*p*<0.05) in *CR* with regard to specific digits between HC and TD groups are indicated by the following symbols: α means the index finger, β means the middle finger and δ means the little finger.

In the comparison with the HC group, a greater *CR* of the index finger in TD_I_ was found throughout the holding task (Fig. 5). In addition, a lower *CR* of the ring finger at H_e_1 was noticed in TD_I_. For the drinking task, the TD_T_ group showed a significantly higher *CR* of the index finger than that of the HC group at D_e_1, D_e_2, and D_e_3, but the middle and little fingers showed lower *CR* at D_e_2 and D_e_3 than those in the HC (Fig. 6). The *CR* of the index finger in TD_M_ was also higher significantly than that in the HC at D_e_2 and D_e_3.

## Discussion

### Correlations between the Radial Force of the Thumb and each Finger

During cylindrical grasp, the F*_r_* of the fingers was associated with the F*_r_* of opposing thumb to execute the holding and drinking task. An analysis of the correlation coefficients between the thumb and each finger revealed the roles of the corresponding fingers at the critical events. The correlation pattern in HC revealed that this group tended to grasp by exerting the F*_r_* of the index, middle and ring fingers corresponding to the F*_r_* of the thumb. Controlling the simulator by using these three fingers and the thumb may be the most efficient way to handle the cylindrical simulator. However, the little finger can be seen as an isolated digit owing to no significant correlation was found between thumb and little finger. A previous study showed similar results on examining how healthy young adults hold cylinders [9]. On the other hand, the F*_r_* of the little finger in all TD groups exhibited significant correlations with that of thumb, especially during the holding task. When grasping a cylindrical object, the little finger can be seen as the chief generator of supination torque in previous studies, due to its mechanical advantage because of its long lever arm [7], [10]. TD patients may thus compensate their control of the simulator by utilizing their intact and advantaged little finger, even if it is not a well-controlled digit. Similar findings have also been reported in patients with carpal tunnel syndrome, in which the intact little finger helped to discriminate the texture of objects [11].

Although the current results showed differences between the correlation patterns of HC and each TD group, it was not only the involved digit, but also the adjacent digits that showed the significant correlations with the thumb. For example, the F*_r_* of the index finger in all TD groups demonstrated a high correlation with the thumb, even in TD_I_. Another phenomenon found in TD_T_ and TD_M_, the patients demonstrated the significant correlations between F*_r_* of thumb and that of each finger, which may be referred to the enslaving effect. In the case of TD_T_, the force control of the thumb may be affected by the disorder, which contributes to the enslaving control of other digits. A previous study suggested that the middle finger, in addition to the ring finger, is the most enslaving digit, and also that the exertion of the index finger contributes to the obvious enslaving force of the adjacent middle finger [12], which may result in the pattern of TD_M_.

Generally speaking, the altered force correlation pattern implies that the different single triggering digit may cause the varied strategies force exertion of fingers corresponding to the force exertion of the thumb. Accordingly, the whole digits of a hand were regarded as an organ of accomplishment to execute the grasping functions, either adopting the compensatory strategies in patients with single triggering digit. Further research works may be needed to follow the correlation among digits after treatment to evaluate whether the compensatory strategies still exist.

### Resultant Forces and Contributions of Digits

During the holding task, only TD_I_ showed significant results with regard to a higher *RF* of the index finger and a higher *CR* of the index finger and lower *CR* of middle finger. When performing the drinking task, a higher *CR* of the index finger was noticed in both TD_T_ and TD_M_ groups compared to the HC during drinking. As seen in TD_T_, TD_I_, or TD_M_, the index finger which plays an important role in hand function may cause significant interruptions in the coordination of digit forces [13]. However, the increase or decrease in *RF* or *CR* did not correspond with the affected digit. A greater force or higher contribution of the index finger was found in several cases in certain TD groups, and this may be because the index finger is usually the best-controlled digit among all fingers [14]. The compensatory patterns adopted by the TD groups may be developed from daily experience to carry out more competent hand manipulations. Nevertheless, the results showed that the total grasping force was not significantly altered in the TD groups. Overall, it can be seen that the central controller system organized the force output of other digits to minimize disturbances by the altered digit force [7], [9].

According to the *CR* results obtained during the drinking task, the middle, ring and little fingers worked in a similar way and tended to form a particular group to couple with the index finger and thumb (Fig. 6). The possible mechanism underlying this is the natural grasping configuration in which the contact positions of the index finger and the group of middle, ring and little fingers were separated by the thumb. When rotating the simulator in the pronation direction during the first phase, the increase in the *CR* of the thumb was associated with the decreasing *CR* of the middle, ring and little fingers, until the simulator was held in a horizontal position. The reverse pattern was then seen in the third phase. This finding not only showed that the modulation of digit force depended on the task [7], [10], [15], but also provided details of the roles of the digits when naturally grasping during dynamic manipulation. The index finger exerted a relatively constant *CR* throughout the drinking task, different to findings of previous reports which showed that the force of the index finger increased to generate pronation torque [4], [7], [10]. These inconsistent results may be due to the configuration of the digits during natural grasping, and the fact that dynamic performance was analyzed in the current study, rather than the planar and static grasping that was examined in the previous studies.

In summary, the abnormal higher force exertion of index finger may contribute to overuse injury after the compensative or repetitive uses. Hence, the reeducation of applied digit force during five-digit manipulation tasks should be considered in the treatment protocol. The biofeedback interface thus may be utilized in training the digit force control in the future.

### The Symptoms of Trigger Digit with Respect to Force Coordination during Grasping

During maximal strength or power grasping tests [2], large flexion angles of the distal and proximal interphalangeal joints were required, which induced triggering phenomena in TD patients. However, the patients in this study did not report triggering or painful sensations when performing the tasks. This may be due to the small range of flexions of the digital joints when grasping the simulator, so that the swollen sheath of tendon was not irritated [16]. The effect of pain can thus be eliminated from the possible causes of the altered patterns of digit force found in this study.

The force patterns found among the TD patients in this work may also have been affected by their daily experience of triggering phenomena. Once triggering has been initiated, more extensor tendon force is required to carry out catching [17]. Although the process of catching may be imperceptible during digit flexion, the resistance that occurs during tendon gliding may compel the flexor to increase the applied force. Another reason for the altered force patterns found in the TD groups may be the patients’ awareness of the joint range at which triggering would be initiated. In order to avoid this and complete the task, the patients may thus adopt a more effective strategy of grasping to manipulate the object, resulting in the force patterns seen in this work.

### The Natural Grasping Function

The force patterns of planar grasp have been investigated by a wide range of studies, which provide valuable knowledge about dexterous and complex control of digits forces hat occur during such actions [4], [7], [10], [15], [18]. In the current study the positions of the force transducers on the simulator were adjusted according to the actual grasping configuration used by each individual patient, to minimize any constraints to natural grasping. This contributed to the different force patterns found in this work compared to those of previous studies. For example, with regard to the *CR* ranking of the digits, Santello et al. reported that the index finger contributed the most among the fingers, followed by the little finger [4]. In contrast, Zhang et al. found that the middle and ring finger contributed the most force during grasping, providing a combined total of more than 60% of the force applied by all four fingers [8]. Although the *CR* of the index finger in TD_T,_ TD_I_ and TD_M_ were larger than that in HC, the *CR* rank of each digit was kept in the same order. In addition, the control of the simulator in space needed the coordination in digit forces found in this work. Therefore, the synchronizing recording of the accelerometer was used to determine the movement events for the analysis of forces, instead of determining the movement phases during the task based on constant time periods [7] or thresholds of digit force [9]. The results presented in the current work demonstrate the strategies used by TD patients with regard to the applied digit forces when dynamically manipulating a cylindrical simulator.

### Limitations

Although the force patterns used by TD patient during functional grasping were successfully examined in this work, there are several limitations that should be noted. First, the cause-effect of the altered patterns found in this work in relation to the focal hand disorder could not be verified by the current findings, and a further, longitudinal study will be needed to examine the effects of pulley release on the pattern of digit forces [19]. A second limitation is that only the digits forces during natural grasping were recorded, and no data was obtained with regard to the applied torque on the simulator. In future work, the spatial positions of the cylinder and transducers should be recorded in order to compute the torque equilibrium dynamically, as this would provide more information about the role of each digit.

## Conclusion

This study investigated the digit forces applied by HC and TD patient with single digit involvement during functional tasks. The altered force patterns were noticed in TD groups. Further, the TD patients seemed to grasp with greater digit forces or more contributions, especially the index finger in TD_T_, TD_I_ and TD_M_. However, the affected digit seemed not to exhibit the particular force correlation and applied force. Instead, the force patterns of TD patients found in this work may relate to the importance of the affected digit in specific tasks.

In conclusion, even if only one digit was affected, the subtle force coordination of all the digits was altered during simple tasks among the TD patients. This study provides the information to further comprehend the possible injuries secondary to the altered finger coordination and also to adopt suitable treatment strategies in the future studies.
